# Acupuncture for post-chemoradiotherapy complications in lung cancer with bone metastasis: a case report and literature review

**DOI:** 10.3389/fonc.2026.1684346

**Published:** 2026-05-25

**Authors:** Kaihua Song, Yang Shen, Xiazhen Xu, Chenxin Jiang, Shouhai Hong

**Affiliations:** 1 The First Clinical Medical College, Zhejiang Chinese Medical University, Hangzhou, Zhejiang, China; 2 The First Affiliated Hospital of Zhejiang Chinese Medical University (Zhejiang Provincial Hospital of Chinese Medicine), Hangzhou, Zhejiang, China

**Keywords:** Acupuncture, cancer pain, chemotherapy-induced peripheral neuropathy, lung cancer with bone metastasis, radiotherapy-related complications

## Abstract

Despite significant advances in oncologic therapeutics, chemoradiotherapy-induced complications, including chemotherapy-induced peripheral neuropathy (CIPN) and hip dysfunction, remain clinically challenging to manage with pharmacological interventions alone. This report presents the case of a 52-year-old male with a history of right-sided lung cancer resection performed more than three years previously and confirmed bone metastasis for over two years. Following metastasis to the right ilium and acetabulum, the patient developed pain, numbness, gait disturbance, and impaired mobility in the right lower extremity. The symptoms deteriorated following local radiotherapy. Marked symptomatic improvement was observed following three courses of acupuncture treatment. At baseline, the patient was diagnosed with Grade III CIPN, with a lower extremity pain Visual Analog Scale (VAS) score of 7.8 and a Harris Hip Score (HHS) of 35. After treatment, CIPN severity improved to Grade I, the VAS score decreased to 3, and the HHS increased to 80. The patient’s Physical Component Summary (PCS) score increased from 28.6 to 72.3, and the Mental Component Summary (MCS) score improved from 34.2 to 78.8. No adverse events were reported throughout the treatment period. The patient reported high satisfaction with treatment outcomes and ceased all analgesic medications. The patient continues to receive maintenance acupuncture to sustain therapeutic benefits and mitigate long-term chemoradiotherapy-related sequelae.

## Introduction

Lung cancer represents the most prevalent malignancy globally, with an estimated 2.1 million new cases and 1.76 million deaths occurring annually (1). The majority of lung cancer-related deaths are attributable to distant tumor metastasis (2). Bone is the most common site of distant metastasis in patients with lung cancer (3, 4). Skeletal metastases affect over 400,000 individuals annually in the United States. Intraosseous tumor progression leads to a spectrum of severe skeletal-related events (SREs), including pain, hypercalcemia, anemia, increased infection risk, pathological fractures, spinal cord compression, spinal instability, and mobility impairment; all of these complications adversely affect patients’ functional status, health-related quality of life, and overall survival (3, 5, 6).

Substantial preclinical and clinical research has elucidated the core molecular mechanisms underlying lung cancer bone metastasis. For instance, Yang et al. (7) reported that formin-like protein 1 (FMNL1) is highly expressed in lung cancer bone metastatic lesions, and that FMNL1 promotes bone metastasis via mediating the transforming growth factor-β1 (TGF-β1)/Smad signaling pathway. In another pivotal study, Chen et al. (8) investigated semaphorin 4D (Sema4D) and demonstrated that hypoxia upregulates HIF-1α, which binds to Sema4D to modulate its secretion; this process in turn inhibits osteoblast differentiation and drives osteolytic bone metastasis in lung cancer.

Pain is one of the most prevalent and debilitating symptoms in patients with malignant tumors (9). Cancer pain can originate from the primary tumor, its metastatic lesions (e.g., bone, soft tissue, visceral organs), and cancer-directed anti-tumor therapies (10). Although pharmacologic analgesia remains the cornerstone of cancer pain management, its clinical efficacy is frequently limited, and its use is associated with a high incidence of dose-limiting adverse effects (9). Approximately one-third of patients treated with chemotherapy develop chemotherapy-induced peripheral nerve injury (11, 12). The symptoms mainly manifest as paresthesia, such as numbness, tingling, or pain in the hands and feet. CIPN is primarily a distal neuropathy syndrome caused by damage and inflammation (13–15). To date, no curative treatments exist for CIPN; conventional agents including opioids and serotonin–norepinephrine reuptake inhibitors (e.g., duloxetine) yield limited efficacy and are associated with intolerable side effects (16, 17). Radiotherapy is an important modality for treating tumors, but radiation therapy induces bone fragility and bone collapse, including bone and metastatic necrosis, and sometimes results in pathological dislocation of the hip joint (18).

Acupuncture, a complementary and alternative medicine developed in traditional Chinese medicine, influences the body by inserting needles into specific points (acupoints) on the human body and is considered an effective adjunct to conventional pharmacological treatments for many cancer-related symptoms and medical conditions (19). The National Comprehensive Cancer Network (NCCN) Clinical Practice Guidelines in Oncology for Adult Cancer Pain recommends acupuncture as a comprehensive treatment option to be used in conjunction with pharmacological therapy (9). The results of a randomized controlled trial showed that women with CIPN after receiving adjuvant taxane therapy for breast cancer experienced clinically and statistically significant improvements in neuropathic symptoms after an 8-week acupuncture treatment regimen (20). In a qualitative report on the feasibility trial of acupuncture in cancer patients undergoing radiation therapy, participants generally indicated that they benefited from acupuncture, believing it brought multiple advantages (21). However, studies on acupuncture treatment for patients with complex symptoms such as pain, soreness, numbness, and other discomforts resulting from lung cancer metastasis to the ilium and acetabulum have not been reported.

In addition, when performed by qualified practitioners, acupuncture is considered a safe therapy for cancer patients and cancer survivors (22). Mild adverse events like dizziness, fatigue, and nausea may appear and be improved without any extra measures. A cumulative review included a total of 715 adverse events, and the results showed that the risk of serious adverse events related to acupuncture is very low, with an estimated incidence of 0.05 cases per 10,000 treatments and 0.55 cases per 10,000 patients (23).

Herein, we present a clinical case of a patient with lung cancer who developed a complex symptom cluster of pain, aching, and numbness following iliac and acetabular metastasis, with further symptom exacerbation after local palliative radiotherapy. This case report was prepared and reported in strict accordance with the 2014 CARE Guidelines for clinical case reports (24).

## Case description

The patient was a 52-year-old male with a 3-year history of right-sided lung cancer post-radical resection and a 2-year history of radiologically confirmed bone metastases. He presented to the Department of Acupuncture at our institution on October 21, 2024, with a 12-month history of progressive right hip pain, right lower extremity aching, numbness, and severe functional limitation of the right hip joint.

On April 2, 2021, the patient presented for follow-up after an incidental pulmonary nodule was detected on a routine physical examination 12 months prior. On April 5, 2021, he underwent video-assisted thoracoscopic surgery (VATS) radical resection of the right lung cancer, with a ~2.5 cm nodule resected from the right upper lung lobe. Postoperative pathology revealed a central adenosquamous carcinoma of the right lung, measuring 4.533cm, with no definite pleural invasion, ALK-negative, and metastasis observed in some lymph nodes. Genetic testing identified an EGFR Ex19del mutation. From May 10 to July 12, 2021, he completed 4 cycles of adjuvant chemotherapy (21-day cycle interval) with the TP regimen: paclitaxel 270 mg intravenously (IV) on Day 1, plus cisplatin 40 mg IV on Days 1–3 of each cycle. From August 4, 2021, to December 2024, he received treatment with Tagrisso (a targeted therapy drug for EGFR mutations) at 80mg, Oral administration, once daily. In December 2024, he was switched to furmonertinib mesylate (Aifusha Mesylate) 40 mg orally once daily, and this regimen was maintained throughout the acupuncture intervention period and follow-up. In February 2023, the patient developed soreness, pain, and numbness in the right lower extremity, with CT and MRI suggesting possible bone metastases. From February 28, 2023, he underwent palliative radiotherapy to metastatic lesions in the right hip, with the right ilium and acetabulum as the target volumes. The prescribed radiotherapy doses were: PTVDT 2000cGy/5f and PGTV 3500cGy/5f. Baseline imaging prior to acupuncture intervention was performed to confirm stable systemic tumor burden: a chest CT obtained on August 2, 2024, demonstrated multiple osteolytic lesions in the thoracic vertebrae, as well as an increased volume of metastatic lesions in the right ilium and acetabulum. A pelvic MRI obtained on September 9, 2024, further confirmed interval progression of bone metastatic lesions in the right ilium, acetabulum, and ischium relative to prior imaging studies. Despite consultations at multiple orthopedic clinics and intermittent use of ibuprofen, his symptoms persisted and progressively worsened. The patient developed secondary depressive symptoms due to refractory pain. On October 21, 2024, he presented to our department with an abnormal gait and restricted movement of the right hip joint. After relevant physical and imaging examinations, other causes such as lumbar disc herniation were ruled out.

The patient had no significant past medical history of hypertension, diabetes mellitus, dyslipidemia, coronary artery disease, chronic obstructive pulmonary disease, chronic liver or kidney disease, or other chronic comorbid conditions. He had a 30-pack-year smoking history and ceased all tobacco use following his lung cancer diagnosis in 2021. Baseline and serial laboratory tests (including complete blood count, serum electrolytes, liver function, and renal function) were performed throughout the intervention period; all results remained within the institutional normal reference ranges, with no acute laboratory abnormalities identified that could confound symptom progression or treatment response.

At baseline, the patient was assessed as having Grade III CIPN per the National Cancer Institute Common Terminology Criteria for Adverse Events (NCI-CTCAE), Version 5.0. His right lower extremity pain had a baseline Visual Analog Scale (VAS) score of 7.8, and his right hip joint function had a baseline Harris Hip Score (HHS) of 35. Baseline health-related quality of life was assessed via the 36-Item Short Form Health Survey (SF-36), with a Physical Component Summary (PCS) score of 28.6 and a Mental Component Summary (MCS) score of 34.2.

### Acupuncture treatment regimen

**Figure 1:**
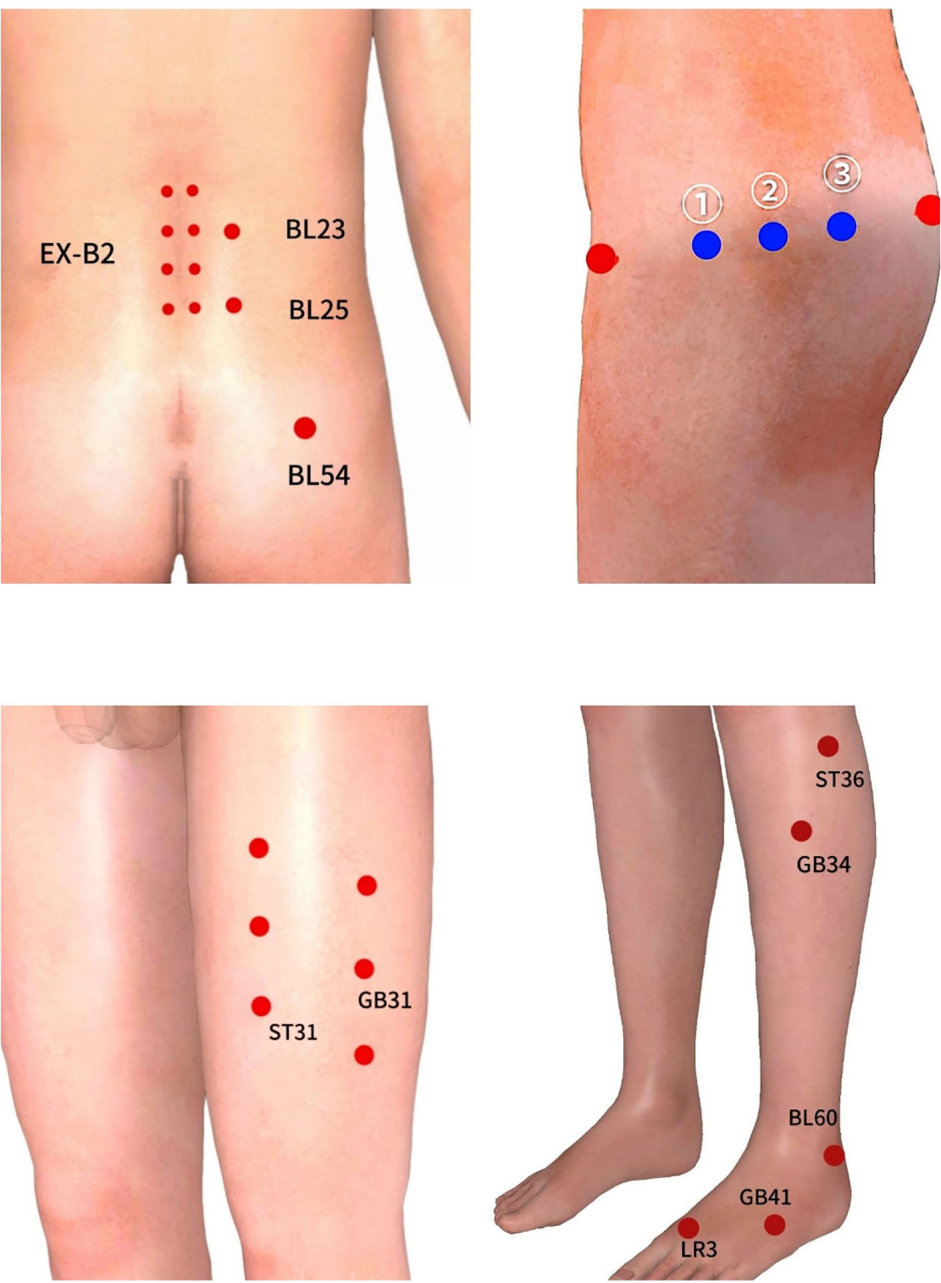
Schematic diagram of acupuncture points.

Following completion of the 5-fraction radiotherapy course, the patient’s symptoms persisted and progressively worsened, with no symptomatic relief achieved despite specialist orthopedic care. He therefore initiated standardized acupuncture treatment at our Department of Acupuncture. Acupoints for electroacupuncture were selected as follows: right-sided lumbar Jiaji (EX-B2) points at L2–L5; Ashi points in the gluteus maximus muscle (3 points selected at equal intervals along the middle three-fifths of the line connecting the posterior superior iliac spine and anterior superior iliac spine); line needling along the Gallbladder Meridian of the thigh (3 cun proximal and 3 cun distal to Fengshi [GB31]); line needling along the Stomach Meridian of the thigh (3 cun and 6 cun proximal to Futu [ST32]); and a line of points from Yanglingquan (GB34) to Zusanli (ST36). Acupoints for manual acupuncture included the following right-sided points: Shenshu (BL23), Dachangshu (BL25), Kunlun (BL60), Taichong (LR3), and Zulinqi (GB41). Warm needle moxibustion was administered at the Zhibian (BL54) point. (See **Figure 1**).

Following standard skin disinfection of all acupoint sites, sterile, single-use acupuncture needles (0.25 mm × 40 mm; Jiachen Brand, China) were inserted perpendicularly into each acupoint. Needle insertion depth was adjusted to elicit the characteristic “Deqi” sensation (a composite of soreness, numbness, distension, and heaviness radiating from the acupoint) for each needle. Following confirmation of Deqi, a low-frequency electroacupuncture device (SDZ-IIB Handheld Electroacupuncture Apparatus; Huatuo Brand, Suzhou Medical Supplies Factory Co., Ltd., Suzhou, China) was used to deliver continuous wave stimulation at a fixed frequency of 2 Hz, with current intensity adjusted between 0.1 mA and 1.0 mA based on the patient’s individual tolerance threshold. For the Zhibian (BL54) point, a 0.3 mm × 75 mm acupuncture needle was used for deep insertion, with the depth adjusted until a radiating sensation was elicited in the ipsilateral lower extremity. All needles were retained for 30 minutes. Concurrent infrared irradiation was applied to the patient’s right hip region throughout the needle retention period, resulting in a total treatment duration of 30 minutes per session. Acupuncture sessions were administered 3 times per week, with each treatment course defined as 4 consecutive weeks of intervention. The patient completed a total of 3 full treatment courses, with maintenance treatment initiated after significant symptomatic improvement was observed.

### Outcomes measurement

Validated, standardized patient-reported and clinician-rated assessment tools were used to evaluate clinical outcomes at baseline and after the completion of each 4-week treatment course:

#### Visual analog scale

The VAS was used to assess the intensity of the patient’s right lower extremity cancer-related pain. The scale comprises a 10-cm horizontal line, with the left anchor labeled “no pain (0)” and the right anchor labeled “worst imaginable pain (10)”. The patient was instructed to mark a point on the line corresponding to their self-perceived pain intensity at the time of assessment. The VAS is a well-established, valid, and highly sensitive tool for the assessment of acute and chronic cancer pain, with extensive psychometric validation in oncology populations (25).

#### Harris hip score

The HHS is a clinician-administered scale specifically developed to evaluate hip joint function and pain-related disability. The scale consists of 4 domains: pain, daily living function, hip deformity, and hip range of motion, with a maximum total score of 100 points. Higher total scores correspond to superior hip function and less pain-related disability. The HHS has well-documented reliability and validity, and is the most widely used scale for evaluating treatment outcomes in patients with hip joint pathology, including oncology-related hip dysfunction (26).

#### Short form-36 health survey

The SF-36 was used to comprehensively evaluate the patient’s health-related quality of life (HRQoL). The survey covers 8 core health domains: Physical Functioning, Role-Physical, Bodily Pain, General Health, Vitality, Social Functioning, Role-Emotional, and Mental Health. These 8 domains are aggregated into 2 summary component scores: the PCS and the MCS. Higher scores on both the PCS and MCS reflect better physical and mental HRQoL, respectively. The SF-36 is the most widely used generic HRQoL assessment tool globally, with well-established reliability and validity across diverse clinical populations, including patients with cancer (27).

#### National cancer institute common terminology criteria for adverse events

The NCI-CTCAE is the global gold standard for grading and reporting adverse events in oncology clinical research and practice. It stratifies CIPN severity from Grade 1 (mild) to Grade 5 (death related to adverse event), based on clinical signs and symptoms, and the impact of neuropathy on the patient’s activities of daily living and functional status (28).

This combination of validated, multi-dimensional assessment tools enabled a comprehensive, quantitative evaluation of the efficacy of the acupuncture intervention across pain, function, HRQoL, and treatment-related toxicity endpoints.

## Results

**Figure 2:**
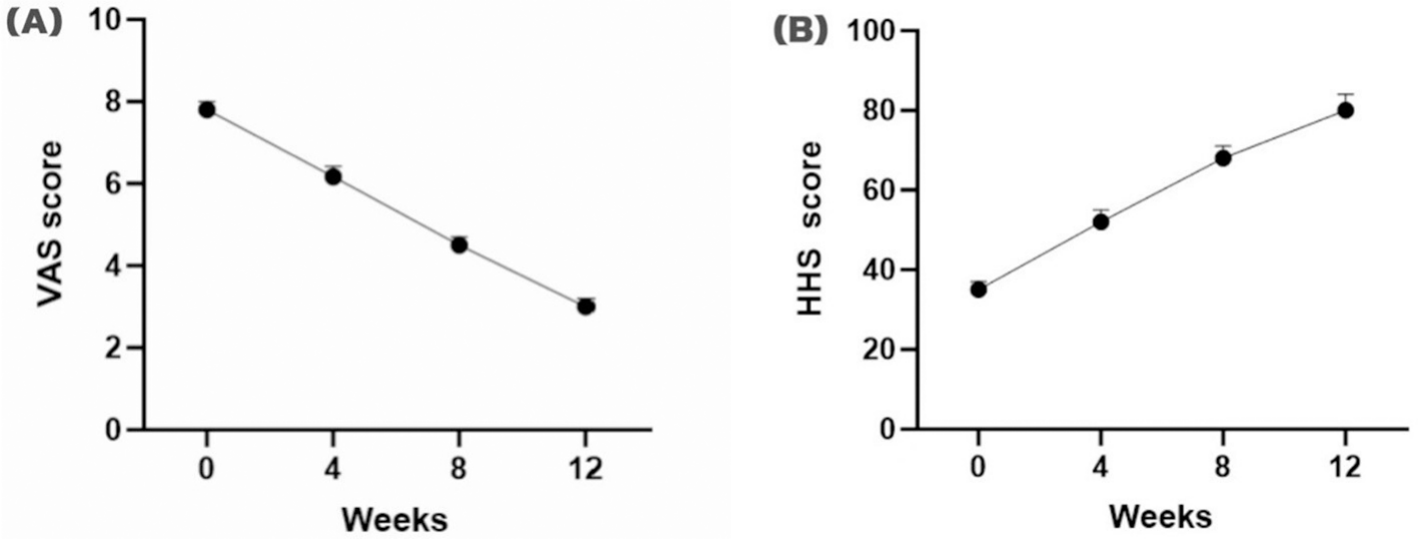
Trajectory of primary outcomes scores over time.

Following completion of the 3 full acupuncture treatment courses, the patient demonstrated clinically meaningful and statistically significant improvements across all primary and secondary outcome measures (**Table 1**). CIPN severity was reduced from Grade III at baseline to Grade I post-intervention; the right lower extremity pain VAS score decreased from 7.8 to 3.0, and the HHS increased from 35 at baseline to 80 post-intervention (**Figure 2**). In parallel with improvements in pain and physical function, the patient exhibited marked improvements in mood, mental state, and overall HRQoL. The patient’s SF-36 PCS score increased from a baseline of 28.6 to 72.3 post-intervention, while the MCS score increased from a baseline of 34.2 to 78.8 post-intervention. No treatment-related adverse events were recorded during the entire intervention period. The patient acknowledged the clinical benefit of acupuncture and reported high satisfaction with the treatment outcomes. The patient completely discontinued all analgesic medications following the initiation of acupuncture treatment, stating: “The effect of acupuncture treatment is very obvious. I stopped taking painkillers after I started receiving acupuncture treatment.” The patient remains on ongoing maintenance acupuncture treatment to sustain the therapeutic benefits and mitigate long-term chemoradiotherapy-related sequelae.

**Table 1:** Comparison of scores before and after treatment.

Assessment metrics	Baseline	Course 1	Course 2	Course 3
VAS	7.8	6.2	4.5	3.0
HHS	35	52	68	80
SF-36 PCS	28.6	45.2	60.8	72.3
SF-36 MCS	34.2	52.6	68.4	78.8

During the post-intervention follow-up period, the patient underwent serial pelvic MRI and chest CT imaging for tumor burden monitoring. Imaging re-evaluation demonstrated no new metastatic lesions, pathological fractures, or spinal cord compression throughout the observation period. The volume and extent of pre-existing bone metastatic lesions remained radiologically stable, with no imaging evidence of tumor progression, regression, or growth inhibition during the entire acupuncture intervention period.

### Literature review

We conducted a literature search in PubMed using the keywords “acupuncture,” “moxibustion,” or “electroacupuncture” in combination with “cancer” and “case reports.” A total of 180 English case reports published between 2000 and 2025 were retrieved, from which 9 highly relevant articles were selected for comparative analysis (**Table 2**).

**Table 2:** Characteristics of published case reports on acupuncture for cancer-related symptoms.

Publication tear and authors	Patient age/sex	Disease and metastasis status	Main symptoms and duration	Regions and acupoints	Treatment cycle and frequency	Treatment outcomes
(29)	68/M	Poorly Differentiated Adenocarcinoma of the Pancreas (multiple metastases in the liver, peritoneum, mesentery, pelvic fascia, and terminal rectum)	The epigastric pain has gradually worsened for more than 3 months.	LU9、LI3、HT7、SI3、PC7、TE3、ST34、SP3、BL65、KI3、GB41、LR3	February 22 to April 19, 2022 (during hospitalization in the terminal stage).	After acupuncture, the interval between subcutaneous morphine injections was significantly prolonged, with the average analgesic effect lasting approximately 16 hours.
(19)	66/F	Hepatocellular carcinoma (cT1N0M0 stage) with sacrum (S1) metastasis, invading the right nerve root and the spinal canal at the L5/S1 level, accompanied by lung metastasis.	Neuropathic pain in the right lower extremity (electric shock-like pain).	GB29、GB30、GB34、GB40、BL40、ST36	4 weeks (until the patient’s death), once a week.	The breakthrough pain disappeared completely.
(30)	78/F	Breast Cancer with C4-TH1 Vertebral Metastases and Spinal Cord Compression	Neuropathic pain persists, with frequent episodes of breakthrough pain.	Based on the ganglionic segment principle (corresponding to the spinal cord segments C5-TH1 involved in the pain)、LU8	Only a single acupuncture session, with the needles retained for 30 minutes.	The pain was gradually brought under control, and the daily dosage of opioid medications was gradually reduced.
(31)	28/F	Lymphangioma	Progressive pain, numbness, and weakness in the right lower extremity;12 days.	CV4、CV6、BL23、BL20、KI3、SP9、SP10、PiGen	3 weeks; once a day (except weekends), with a total of 15 sessions.	The clinical symptoms disappeared completely; MRI showed a significant reduction in the mass of the right lower extremity.
(32)	73、64/F	After chemoradiotherapy for endometrial carcinoma	Burning sensation and electric shock-like pain in the left lower extremity,/Pain and weakness in the right lower extremity.	SP06、SP11、SP12、LR04、LR08、LR10、KI03、BL40、GV02、GV03、BL32、BL35、CV4 、CV6	8 weeks, 3 times a week, totaling 20 sessions; 4 weeks, 3 times a week, totaling 10 sessions.	The pain disappeared completely.
(33)	48/M	Multiple myeloma with multiple bone lesions	Pins and needles sensation, numbness, and burning pain in both feet, lasting for more than 4 months.	Ear Acupuncture; LI4、SJ5、LI11、ST40、BaFeng	A total of 14 sessions.	The VAS pain score decreased from 8 to 2; morphine/oxycodone was discontinued, with no recurrence during a 1-year follow-up.
(34)	60–71/F	Advanced gynecological malignancies (such as ovarian cancer), with peritoneal metastasis in some cases	Numbness, burning pain, and tingling in the hands and feet, accompanied by an unsteady gait; symptoms lasting 6–38 months.	CV6、ST36、LI11、Ba Feng、Ba Xie、JingXue	Once a week for 6 weeks → 4 weeks of rest → another 6 weeks, totaling 12 sessions (one patient completed only 7 sessions).	The average pain score decreased from 7.8 to 3, and analgesics were reduced or discontinued.
(35)	64/M	Non-Hodgkin Lymphoma (NHL)	Radiating pain in the left buttock, anterolateral thigh, knee, and lateral lower leg, lasting for 2 weeks.	GB30、Ashi Point	Once a week for 4 weeks, then changed to once every two weeks for 1 month, with a total of 6 sessions.	The severe pain in the buttocks and legs did not recur, and the oral analgesics were gradually reduced and eventually discontinued.
(36)	74/M	After chemotherapy for multiple myeloma (MM)	Pain, numbness, tingling, and weakness in the hands and feet, lasting for 4 weeks.	ST36、SP6、LI4	The frequency was adjusted from once every two days to twice a week, with a total of 15 sessions; subsequent maintenance treatment is administered once a month.	The initial pain score was 8/10. After the 2nd treatment, the pain score decreased to 4/10; after the 15th treatment, the pain score dropped to 0/10, and the symptoms were completely relieved.

The reviewed cases encompass a variety of cancer types, including pancreatic cancer, hepatocellular carcinoma, breast cancer, gynecological malignancies, lymphoma, multiple myeloma, and lymphangioma. These reports highlight the application of acupuncture in managing cancer-related pain, neuropathic pain induced by chemotherapy or radiotherapy, and other treatment-related complications such as peripheral neuropathy and functional impairment.

Acupuncture has been frequently reported to alleviate neuropathic pain, including breakthrough pain and CIPN. For instance, Liu et al. (29) observed prolonged intervals between morphine injections in a pancreatic cancer patient, while Bao et al. (33) and Wong & Sagar (34) documented significant reductions in pain scores and opioid use in patients with multiple myeloma and gynecological cancers, respectively. Patients often reported enhanced mobility, mood, and overall quality of life. Su (19) noted that home-based acupuncture eliminated breakthrough pain and improved daily functioning in a patient with hepatocellular carcinoma and sacral metastasis. The acupoint selection protocol includes not only acupoints based on the traditional meridian theory but also those selected under the guidance of neuroanatomy (30). In clinical practice, the combination of “local acupoints + distal acupoints” is often adopted to simultaneously improve local symptoms and regulate the balance of the body’s overall functions.

In the aforementioned cases, acupuncture was used as an adjuvant treatment. It could reduce patients’ dependence on medications and alleviate drug side effects, thereby providing support for the multimodal treatment of cancer. Generally, patients tolerated acupuncture well, with no serious adverse events reported. Some cases showed therapeutic effects after several treatment sessions, while others required continuous treatment for several weeks to achieve stable outcomes.

## Discussion and conclusion

This case report demonstrates the potential efficacy and safety of acupuncture as a complementary and integrative medicine modality for the management of a complex, refractory symptom cluster—including cancer-related bone pain, CIPN, gait disturbance, and hip dysfunction—in a patient with lung cancer and bone metastasis following chemoradiotherapy. Despite receiving standard orthopedic care and intermittent analgesic pharmacotherapy, the patient’s pain, functional impairment, and CIPN persisted and progressively worsened; sustained, stepwise clinical improvement was observed only after the initiation of the standardized acupuncture intervention. All quantitative outcome measures demonstrated sustained, stepwise improvement throughout the 12-week intervention period, providing supportive (albeit not definitive) evidence that acupuncture was the primary driver of the observed therapeutic benefits. Following completion of the 3 full treatment courses, all clinical outcome indicators showed clinically meaningful and statistically significant improvement; these improvements were accompanied by complete discontinuation of all analgesic medications, with no treatment-related adverse events recorded during the intervention.

The growth of tumors in the bones can lead to pain, bone fractures, spinal cord compression, spinal instability, and decreased mobility, all of which can impair the patient’s functional status, quality of life, and survival rate (3, 5, 6). Opioid analgesics are often accompanied by adverse reactions such as constipation, nausea, and vomiting (37). Non-steroidal anti-inflammatory drugs (NSAIDs) can increase the risk of gastrointestinal bleeding (9). During the chemotherapy process, the combination use of paclitaxel and cisplatin may lead to neuropathy in approximately 70% of patients (38). Cisplatin can cause sensory neuropathy due to its effects on the dorsal root ganglia (DRG) (39). When taxane-based drugs are used in combination with cisplatin, the toxicity is more severe. Paclitaxel can induce mainly sensory axonal neuropathy and painful distal sensory neuropathy, including mechanical hyperalgesia, cold hyperalgesia, continuous burning pain, tingling, and numbness (38). After the patient in this case developed lung cancer metastases, he underwent five sessions of radiation therapy, which exacerbated his symptoms. The acetabulum, as a weight-bearing joint structure, can become fragile and collapse due to radiation therapy, including bone and metastatic necrosis, and sometimes even lead to pathological dislocation of the hip joint (18).

The therapeutic effects of acupuncture observed in this case may be explained by several mechanisms. Electroacupuncture can inhibit substances such as Substance P (SP) and Interleukin-1β (IL-1β) in the spinal cord, effectively blocking cancer pain. It activates A-β, A-δ, and C afferent fibers, transmitting signals to the sensory-motor cortex of the brain, and accelerating nerve regeneration (40–42). Acupuncture stimulates nociceptive Aδ and C fibers, modulates neuronal activity in the spinal dorsal horn after chemotherapy, and improves symptoms of peripheral neuropathy, including pain, burning sensation, numbness, hyperesthesia, hyperalgesia, impaired temperature discrimination, and gait disturbances (43). In this case report, the Dorsal rami of L2-L5 (EX-B2) and the gluteus maximus trigger band share the same metameric innervation with the ilium-acetabular lesion; electro-stimulation at 2 Hz thus produces immediate segmental inhibition of nociceptive input (41). The Ashi points in the gluteus maximus muscle, and BL 54 are located in the region where the sciatic nerve runs. These points can directly stimulate the afferent fibers of the sciatic nerve and activate Aδ and C fibers. Alleviate paclitaxel-induced neuropathic pain through antioxidant pathways (44), and enhance the availability and utilization of brain-derived neurotrophic factors (45). In corresponding animal models, electroacupuncture significantly alleviated inflammatory, neuropathic, cancer-related, and visceral pain. (41).

In addition, acupuncture can increase blood perfusion in the fingertips, improve terminal circulation, nourish neurons, and facilitate the repair of neuropathy (46). It has been proposed that acupuncture activates GABAergic, serotonergic, and adrenergic neurotransmission by modulating neurotrophic factors and nerve growth factors, and deactivates the hypersensitization of sensory neurons (47). In this case report, the Gallbladder Meridian and Stomach Meridian run along the lateral and anterior aspects of the lower extremities, covering the lateral femoral cutaneous nerve, common peroneal nerve, tibial nerve, and other nerves. Their distribution highly coincides with the symptom-affected areas of CIPN. Meanwhile, stimulation of distal acupoints such as LR3, GB41, and BL60 can activate multiple brain regions, including the Nucleus Raphe Magnus, Periaqueductal Gray, Anterior Cingulate Cortex, and hypothalamus, thereby regulating pain perception and emotional responses (42). Recent research has shown that acupuncture at the Zusanli (ST36) acupoint has therapeutic effects on multiple systemic diseases. It primarily participates in regulating inflammatory states, oxidative stress homeostasis, immune cell function, hormone secretion, neurotransmitter transmission, and more (48). Jeun et al. reported that acupuncture at Yanglingquan (GB34) can modulate cortical activity in the somatosensory motor area of the human body (49).

Emerging preclinical research has identified multiple biological pathways through which acupuncture may exert anti-tumor, immunomodulatory, and bone-protective effects. In immunocompetent murine models, electroacupuncture has been shown to favorably modulate the tumor immune microenvironment, enhancing anti-tumor immunity by increasing intratumoral infiltration of cytotoxic CD8+ T cells and natural killer (NK) cells, while reducing the abundance of immunosuppressive M2-type tumor-associated macrophages and regulatory T cells (50, 51). Further preclinical studies have demonstrated that acupuncture may regulate the balance between osteoclast and osteoblast activity in bone metastasis, inhibiting osteoclast-mediated bone resorption via downregulation of the RANK/RANKL/OPG signaling pathway, thereby reducing osteolytic bone destruction (52, 53). In addition, preclinical studies have shown that acupuncture mitigates chemotherapy-induced normal tissue toxicity, potentially reducing the incidence of treatment-related adverse events that lead to chemotherapy dose reductions or treatment interruptions (54).

Despite these promising preclinical findings, there is currently no high-quality, large-scale clinical evidence to confirm that acupuncture exerts disease-modifying anti-tumor effects in patients with lung cancer or bone metastasis. The overwhelming majority of clinical trials and systematic reviews of acupuncture in oncology have focused exclusively on symptom management and HRQoL improvement; to date, no studies have demonstrated validated clinical endpoints showing that acupuncture modifies tumor growth, metastasis, or overall survival in human patients with cancer (55, 56). This critical distinction aligns with the recommendations of the National Comprehensive Cancer Network (NCCN) Clinical Practice Guidelines in Oncology, which recommend acupuncture as a non-pharmacological intervention for the management of cancer pain, CIPN, and other treatment-related symptoms, but do not endorse its use as a primary anti-cancer therapy (9).

The key novelty and clinical value of this case report lie in its focus on a complex, multi-factorial symptom cluster that has rarely been addressed in the existing acupuncture literature. While prior studies have primarily focused on acupuncture for CIPN alone (20, 34) or neuropathic pain from metastasis (19, 30), our case highlights its potential dual role in addressing neuropathic and musculoskeletal components simultaneously. The observed functional recovery, as reflected in the HHS improvement from 35 to 80, suggests that acupuncture may contribute to restoring mobility and quality of life even in the context of structural compromise.

This case report provides valuable preliminary clinical evidence for the use of acupuncture as a safe, effective adjuvant intervention for the management of complex, treatment-refractory chemoradiotherapy-related complications in patients with lung cancer and bone metastasis. However, these findings reflect the individualized clinical benefit observed in a single patient and thus have limited generalizability to broader oncology populations. The efficacy of acupuncture for this complex, multi-symptom clinical presentation requires further validation in large-sample, randomized controlled trials.

## Data Availability

The original contributions presented in the study are included in the article/supplementary material. Further inquiries can be directed to the corresponding author.
